# Characterization and differentiation potential of mesenchymal stem cells isolated from multiple canine adipose tissue sources

**DOI:** 10.1186/s12917-021-03100-8

**Published:** 2021-12-18

**Authors:** Usman Rashid, Arfan Yousaf, Muhammad Yaqoob, Evelyn Saba, Muhammad Moaeen-ud-Din, Shahid Waseem, Sandra K. Becker, Gerhard Sponder, Jörg R. Aschenbach, Mansur Abdullah Sandhu

**Affiliations:** 1 Department of Clinical Studies, Faculty of Veterinary and Animal Sciences, PMAS-Arid Agriculture University, Rawalpindi, 46300 Pakistan; 2 Department of Veterinary Biomedical Sciences, Faculty of Veterinary and Animal Sciences, PMAS-Arid Agriculture University, Rawalpindi, 46300 Pakistan; 3 Department of Animal Breeding and Genetics, Faculty of Veterinary and Animal Sciences, PMAS-Arid Agriculture University, Rawalpindi, 46300 Pakistan; 4 ABO Scientific, Rawalpindi, 46300 Pakistan; 5 Institute of Veterinary-Physiology, Freie Universität Berlin, Berlin, Germany

**Keywords:** Mesenchymal stem cell, Adipose tissue, Dog, Adipogenesis, Osteogenesis

## Abstract

**Background:**

Mesenchymal stem cells (MSCs) are undifferentiated cells that can give rise to a mesoderm lineage. Adipose-derived MSCs are an easy and accessible source for MSCs isolation, although each source of MSC has its own advantages and disadvantages. Our study identifies a promising source for the isolation and differentiation of canines MSCs. For this purpose, adipose tissue from inguinal subcutaneous (SC), perirenal (PR), omental (OM), and infrapatellar fat pad (IPFP) was isolated and processed for MSCs isolation. In the third passage, MSCs proliferation/metabolism, surface markers expression, in vitro differentiation potential and quantitative reverse transcription PCR (*CD73*, *CD90*, *CD105*, *PPARγ*, *FabP4*, *FAS*, *SP7*, *Osteopontin*, and *Osteocalcin*) were evaluated.

**Results:**

Our results showed that MSCs derived from IPFP have a higher proliferation rate, while OM-derived MSCs have higher cell metabolism. In addition, MSCs from all adipose tissue sources showed positive expression of CD73 (NT5E), CD90 (THY1), CD105 (ENDOGLIN), and very low expression of CD45. The isolated canine MSCs were successfully differentiated into adipogenic and osteogenic lineages. The oil-red-O quantification and adipogenic gene expression (*FAS*, *FabP4*, and *PPARγ*) were higher in OM-derived cells, followed by IPFP-MSCs. Similarly, in osteogenic differentiation, alkaline phosphatase activity and osteogenic gene (*SP7* and *Osteocalcin*) expression were higher in OM-derived MSCs, while *osteopontin* expression was higher in PR-derived MSCs.

**Conclusion:**

In summary, among all four adipose tissue sources, OM-derived MSCs have better differentiation potential toward adipo- and osteogenic lineages, followed by IPFP-MSCs. Interestingly, among all adipose tissue sources, MSCs derived from IPFP have the maximum proliferation potential. The characterization and differentiation potential of canine MSCs isolated from four different adipose tissue sources are useful to assess their potential for application in regenerative medicine.

## Background

In the last decade, regenerative medicine has gained significant attention in human and veterinary medicine because of its potential role in healing or replacing apoptotic cells. The basic principle of regenerative medicine is to deliver stem cells to target sites that can promote the development of tissues/organs. A stem cells is an undifferentiated cell that is able to proliferate indefinitely and can differentiate into different types of cells. According to the source, stem cells are divided into embryonic stem cells, mesenchymal stem cells (MSCs) and induced pluripotent stem cells (iPSCs), as described by [1]. In canines, MSCs can be isolated from different parts of the body, including adipose tissue [2] bone marrow, umbilical cord [3], and synovial fluid [4]. International Society for Cell Therapy recommended three basic characteristics of the regenerative potential of MSCs, including plastic adhesion, the expression of clusters of differentiation (CD) including CD73, CD90, CD105, and multi-lineage differentiation [5]. Adipose-derived mesenchymal stem cells (AD-MSCs) have attracted attention because they can be easily collected by minimal invasive surgery. Contrasted with bone marrow-derived MSCs (BMSCs); AD-MSCs produce more cells, have a higher proliferation rate, and maintains their phenotype during higher passages [6]. Canine models are an excellent choice for studying various human musculoskeletal conditions because of their common pathological implications and clinical manifestation [7]. Past literature on canine species has proven the use of AD-MSCs for the treatment of osteoarthritis of the elbow [8], coxofemoral [9] and humeroradial joint [10].

**Fig. 1:**
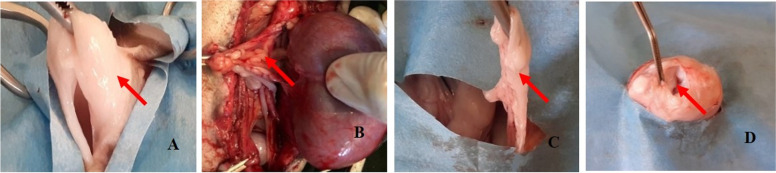
Tissue collection sites of dogs. **A.** Inguinal subcutaneous, **B.** Perirenal, **C.** Omentum fat, **D.** Infrapatellar fat pad

In canines, inguinal subcutaneous adipose-derived MSCs showed a more rapid proliferation rate than omental adipose-derived MSCs; however, no differences were observed in lineage differentiation [11]. Another study on dogs showed that omental adipose tissue can be easily obtained regardless of the animal’s condition, and always has a higher cell yield than subcutaneous adipose tissue [12]. The infrapatellar fat pad (IPFP) is another promising source of MSCs isolation and differentiation, with higher cell yield, colony-forming capacity, proliferation rate, and efficient differentiation to adipocytes as compared to subcutaneous adipose tissue and BM-MSCs [4]. However, most of the available studies in the literature on the isolation/differentiation of MSCs have compared two or three adipose tissue sources, or presents the comparisons between adipose tissue sources and bone marrow. The main objective of this study was to evaluate isolation, characterization, and differentiation efficiency of canine AD-MSCs due to their importance in veterinary medicine and their role as a study model for human musculoskeletal problems (Fig. 1).

## Results

### Isolation, and expansion of canine mesenchymal stem cells

**Fig. 2:**
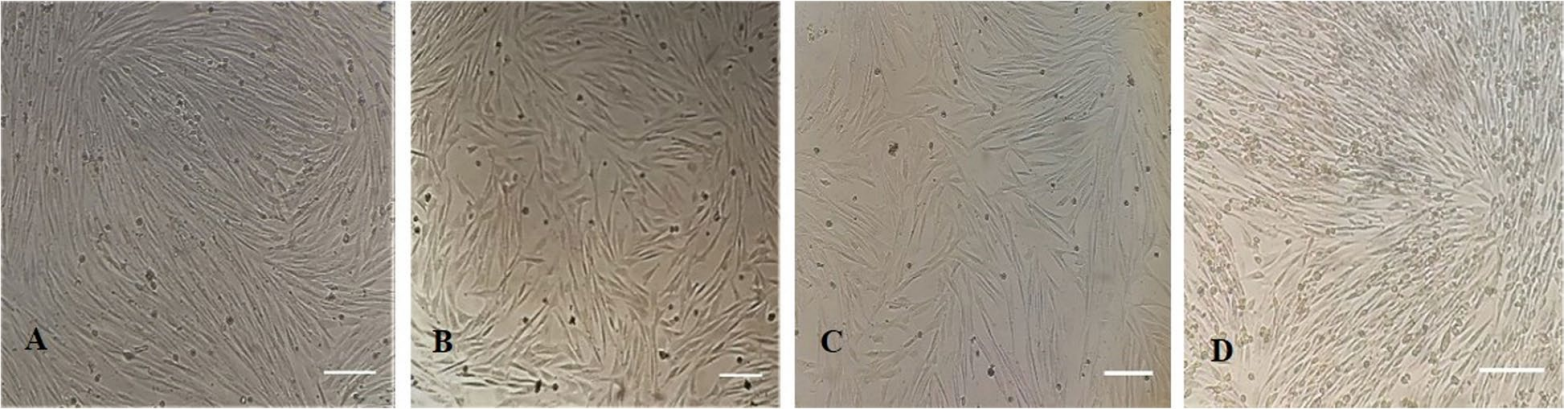
The phenotype of canine MSCs at passage 3 (objective 20x). Panel **A.** Subcutaneous **B.** Perirenal **C.** Omental fat-derived MSCs. All recovered cells have a long spindle-shaped appearence, while, **D.** Infrapatellar fat pad derived MSCs were relatively smaller in size as compared to panel A, B, and C. Scale bar = 20 μm

Stem cells were successfully isolated from SC, PR, OM, and IPFP cultured adipose tissue sources as shown in Fig. 2A-D. MSCs showed a typical fibroblast-like morphology, except for the MSCs recovered from IPFP, the cell size was smaller than that of other AD-MSCs (Fig. 2D). However, all the separated MSCs adhere to the plastic surface, regardless of their source.

### Mesenchymal stem cells doubling time

**Fig. 3:**
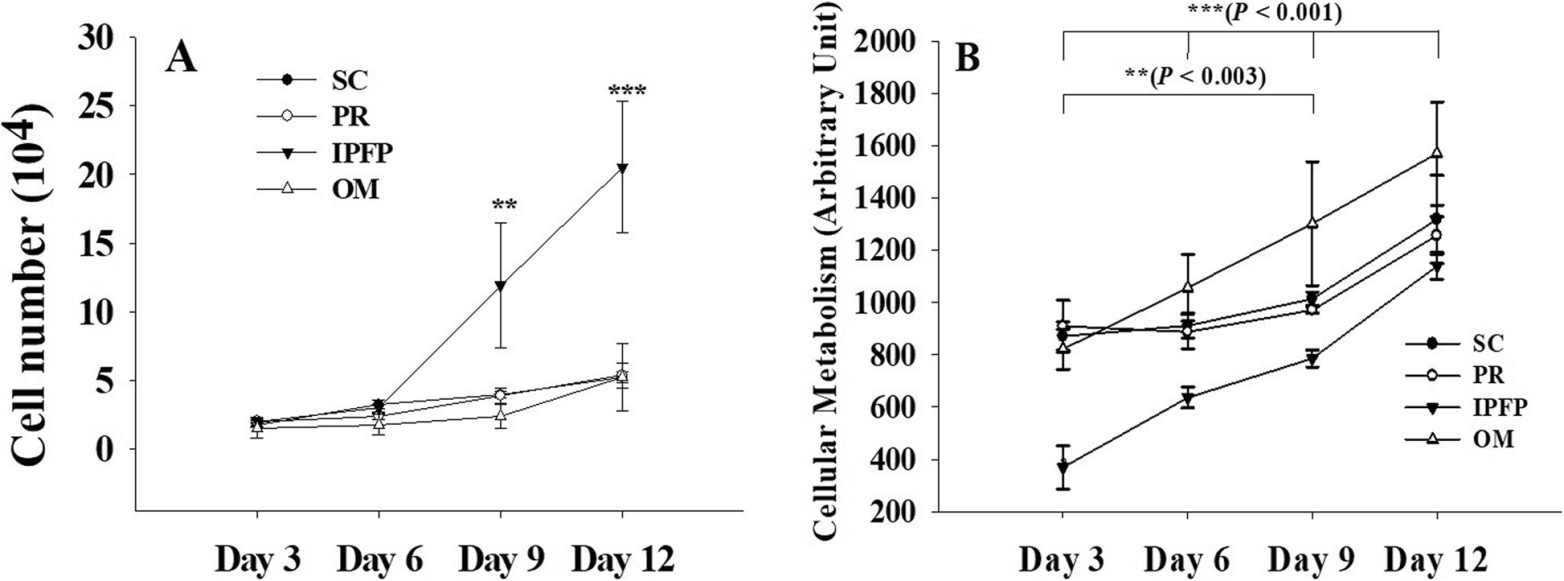
(**A**) Cell doubling time on days 3, 6, 9, and 12. *** *P* < 0.001, ** *P* = 0.004. (**B**) Cellular Metabolism (MTT assay) at day 3, 6, 9 and 12. The data is shown as mean ± SEM. Significance among days, ***P < 0.001, **P = 0.003. Abbreviations: **S/C:** Subcutaneous, **PR:** Perirenal, **OM:** Omentum, **IPFP:** Infrapatellar fat pad

The cell doubling time of MSCs isolated from different sites was evaluated at P3 (Fig. 3A). On the 3rd and 6th day, cells from all sources showed non-significant (*P* > 0.05) change in the proliferation rate. However, the doubling rate of IPFP-derived MSCs increased significantly (*P* < 0.005) on the 9th and 12th days, while the proliferation rate of SC, PR, and OM-derived MSCs did not change significantly.

### Mesenchymal stem cells metabolic assay

The metabolic activity of MSCs on days 3, 6, 9, and 12 was evaluated at P3. Our results showed that OM, SC, and PR derived MSCs have a significantly (*P* = 0.004) higher cellular metabolism compared to IPFP-MSCs. The metabolic activity of all adipose tissue-derived MSCs increased significantly (*P* < 0.003) on day 9 compared to that at day 3. However, on day 12th the cellular metabolic activity remained significant (*P* < 0.001) in comparison with all previous days irrespective of tissue origin (Fig. 3B).

### Immunophenotyping of mesenchymal stem cells

**Fig. 4:**
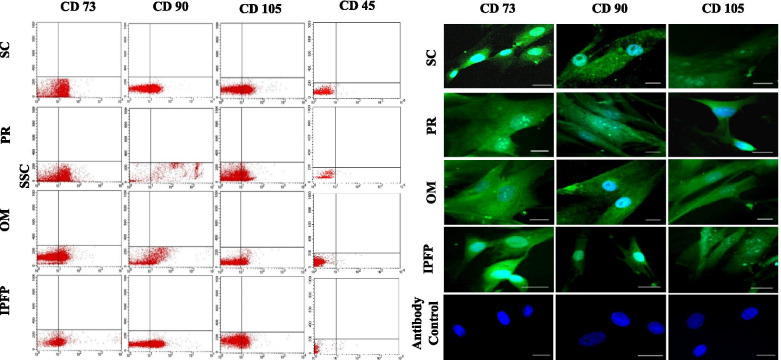
(**A**) Flow cytometric expression of CD73, CD90, CD105, and CD45 in undifferentiated canine MSCs, derived from different fat sources. (**B**) Immunostaining of undifferentiated cMSCs derived from different tissue sources. The cells were stained positive with surface antigens CD73, CD90, and CD105 (green) and nuclear staining (DAPI, blue). Scale bar = 25 μm. Abbreviations: **S/C:** Subcutaneous, **PR:** Perirenal, **OM:** Omentum, **IPFP:** Infrapatellar fat pad

At P3, the cell surface antigen expression of MSCs from all four fat sources was evaluated by flow cytometry and immunofluorescence. Our results revealed that cellular expression percentage of CD73 amounted to 56.2, 45.2, 42.9, 46.7% in SC, PR, OM and IPFP, respectively, whereas the expression percentage of CD90 was 58.9, 53.2, 53.4, and 54.9%, and the percentage of CD105 positive cells was 30.1, 25.8, 29.1, and 27.6%, respectively. The expression of CD45 in SC, PR, OM, and IPFP derived MSCs was 1.2, 1.74, 1.54 and 1.05% respectively, as shown in Fig. 4A. Adipose tissue-derived cells grown in complete LG-DMEM medium were evaluated for well-defined MSCs markers through immunophenotyping and conferred the scattered presence of CD73*,* CD90, and CD105 cell surface markers throughout the cell membrane, as shown in Fig. 4B.

### Adipogenesis

**Fig. 5:**
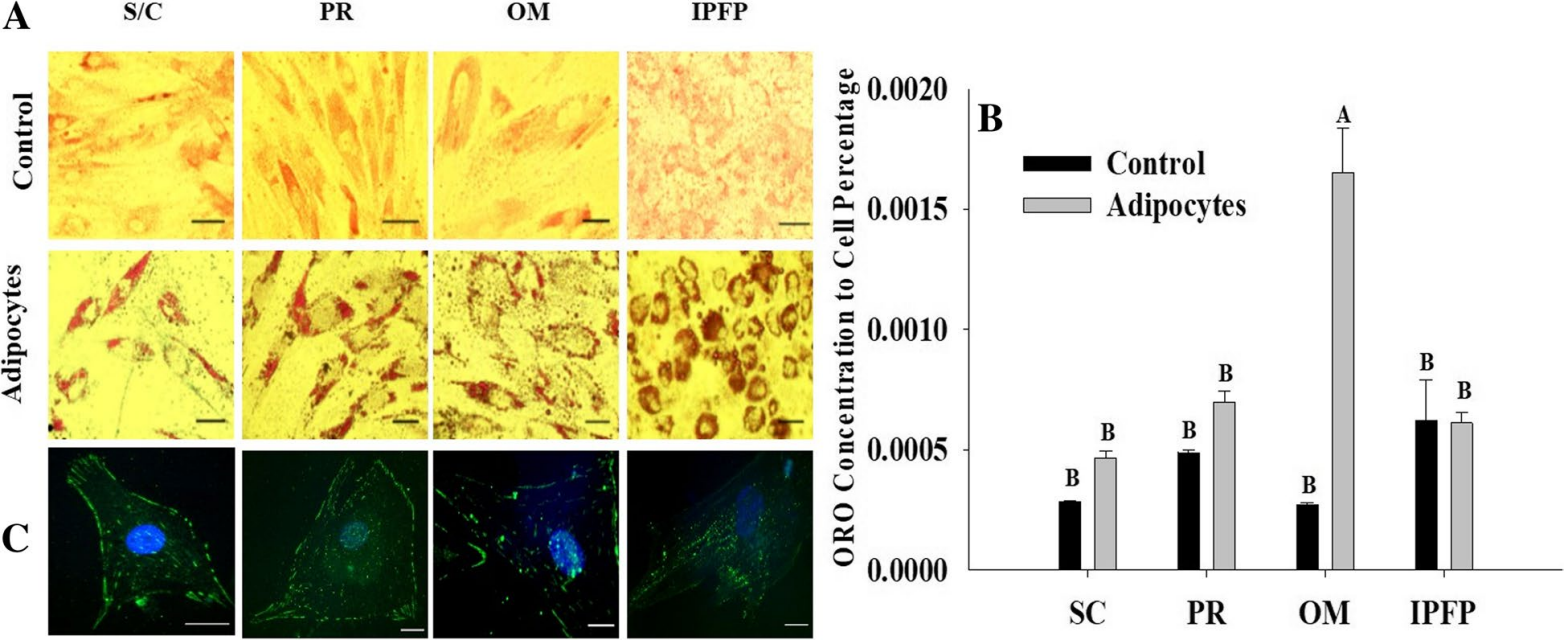
After 7 days of adipogenesis in the adipogenic media the cells were stained with oil-red-O (**A**) control and adipocytes (Objective 20×). Scale bar = 20 μm. (**B**) Quantification of ORO staining between undifferentiated cells (control) and differentiated adipocytes. The data was normalized to the number of cells and expressed as the mean ± SEM. Superscripts letter ^A^ indicates a level of significance *P* < 0.001. (**C**) Immuno-expression of FabP4, cells from all fat sources showed positive expression of FabP4. Scale bar = 25 μm. Abbreviations: **S/C:** Subcutaneous, **PR:** Perirenal, **OM:** Omentum, **IPFP:** Infrapatellar fat pad

To confirm adipogenesis, cells were stained with ORO for the identification of lipid droplets. The stained lipid droplets appeared red under in an inverted light microscope (objective 40×) as shown in Fig. 5A. For quantitative analysis of ORO, the dye was eluted with anhydrous isopropanol and the absorbance was measured spectrophotometrically at 490 nm. OM adipose-derived MSCs showed a significant difference (*P* < 0.001) in the concentration of ORO compared to undifferentiated precursor cells; however, SC, PR, and IPFP-derived MSCs did not develop significant differences in the concentrations of ORO during adipocyte differentiation as shown in Fig. 5B. The differentiated adipocytes showed the presence of FABP4 on the cell membrane and around the accumulating cytoplasmic lipid vacuoles (Fig. 5C).

### Osteogenesis and alkaline phosphatase activity (ALP)

**Fig. 6:**
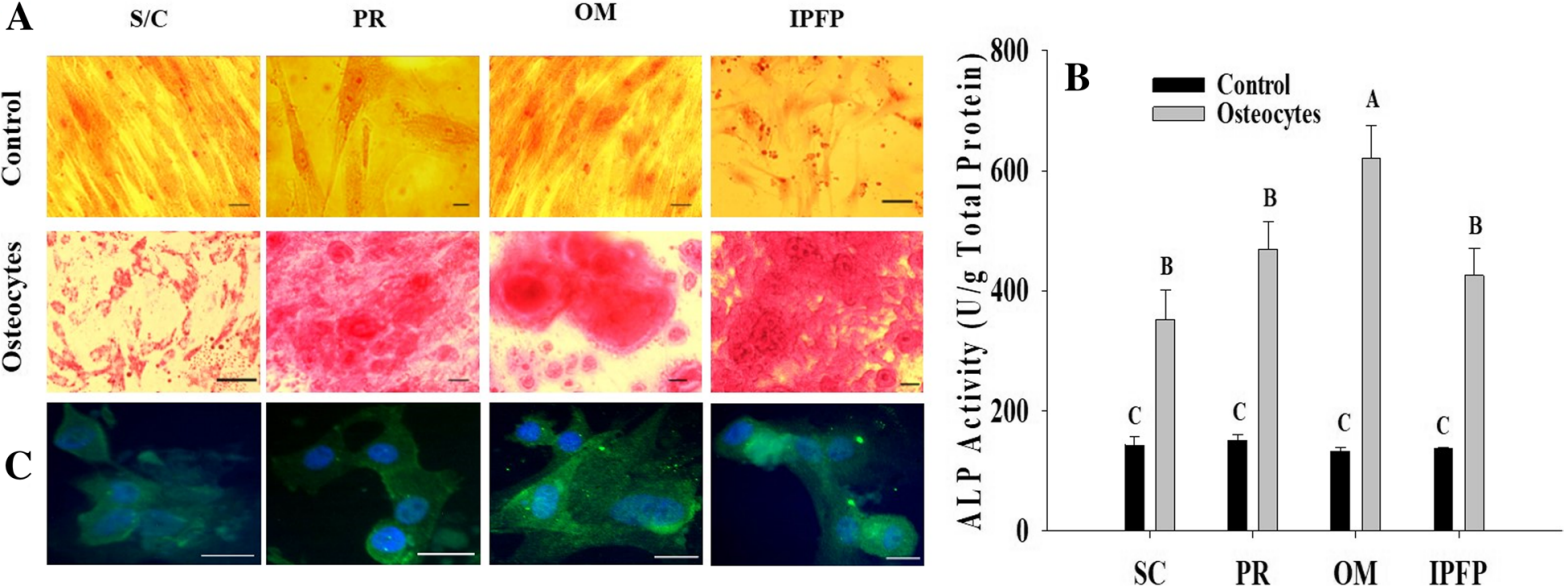
Alizarin red staining of canine MSCs in osteogenic medium after 21 days. (**A**) Control and osteogenic cells show deposition of hydroxyapatite mineral stained red with Alizarin red stain (objective 20×). Scale Bar = 20 μm. (**B**) The ALP activity of differentiated osteocytes compared to undifferentiated control cells. The data was normalized by unit per gram of protein and expressed as mean ± SEM. ^A-C^Different superscripts letters indicate significant differences (*P* < 0.05). (**C**) Immuno-expression of osteopontin in canine MSCs recovered from all four fat sources and kept in osteogenic media showed a positive expression of Osteopontin. Scale bar = 25 μm. Abbreviations: **S/C:** Subcutaneous, **PR:** Perirenal, **OM:** Omentum, **IPFP:** Infrapatellar fat pad

The osteogenic abilities of MSCs isolated from different sites were evaluated by treating them with osteogenic growth media for a period of 21 days and staining with Alizarin Red Stain. The differentiated cells showed positive staining of the extracellular mineral matrix, as shown in Fig. 6A. Alkaline phosphatase activity is considered to be an early sign of osteogenesis. Compared with undifferentiated cells, the ALP activity of osteo-induced canine MSCs was significantly higher (*P* < 0.01). Furthermore, as shown in Fig. 6B, the ALP activity of differentiated cells from OM was higher (*P* ≤ 0.05) than that of cells from SC, PR, and IPFP. Osteocytes differentiated from all MSC sources showed positive expression of *osteopontin* as shown in Fig. 6C, though the antigen was diffusely distributed all over the cells.

### Gene expression studies

**Fig. 7:**
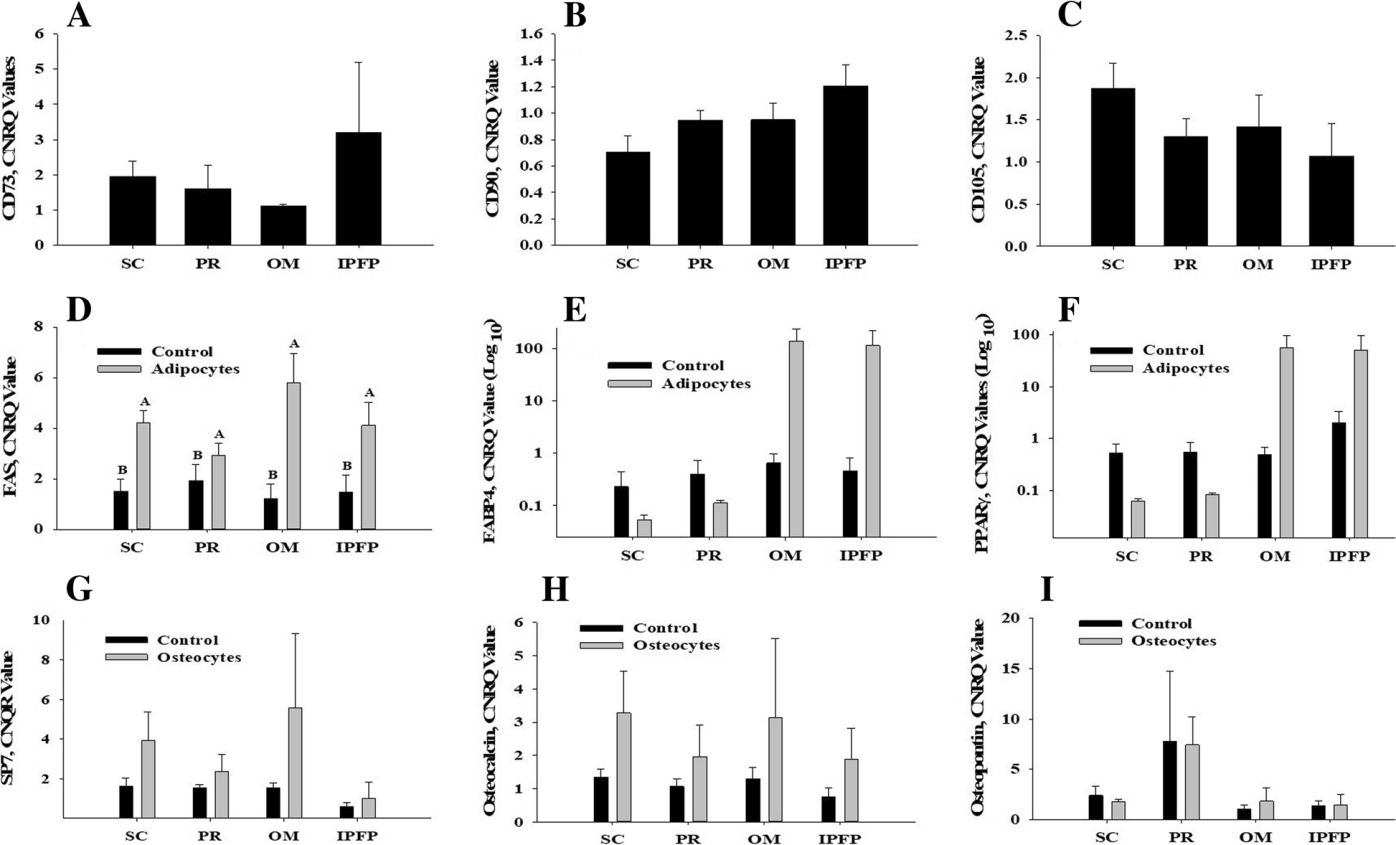
At P3, the MSCs from all four fat sources were evaluated for the relative mRNA expression (CNRQ) of *CD73* (**A**), *CD90* (**B**), and *CD105* (**C**) genes. After 7 days of adipogenesis, the differentiated adipocytes were evaluated for expression of *FAS* (**D**), *FABP4* (**E**), and *PPARγ* (**F**). While, the expression of osteogenic genes *SP7* (**G**), *Osteocalcin* (**H**), *Osteopontin* (**I**) were evaluated in differentiated cMSC after 21 days in osteogenic media. The data was presented as mean ± SEM. ^A,B^Different superscripts letter indicates significant differences within a given adipocyte source (*P* < 0.001). Abbreviations: **S/C:** Subcutaneous, **PR:** Perirenal, **OM:** Omentum, **IPFP:** Infrapatellar fat pad

The gene expression of *CD73, CD90*, and *CD105* was tested in undifferentiated canine MSCs. Our results indicated that regardless of the tissue source the expression of *CD73* (*P* = 0.577)*, CD90* (*P* = 0.113) and *CD105* (*P* = 0.406) were non-significant as shown in Fig. 7A-C. Therefore, the results of qPCR confirmed that the cells used in this study were canine MSCs. Further, the expression of adipogenic markers (*FAS, FABP4,* and *PPARγ*) revealed that, among all fat sources, OM-derived MSCs had significantly (*P* < 0.01) higher expression of *FAS*. In contrast, regardless of the tissue source expression of *FABP4* (*P* = 0.386) and *PPARγ* (*P* = 0.418) did not change significantly during the differentiation process as presented in Fig. 7D-F. However, compared to control cells, adipocytes differentiation from OM and IPFP fat sources tended to show a higher expression of *FABP4* and *PPARγ*. When MSCs were differentiated to osteogenic lineage, they were evaluated for osteogenic markers (*SP7, osteocalcin,* and *osteopontin*) as shown in Fig. 7G-I. Our results showed that, compared with the other four sources, the expression of *SP7* in OM fat-derived MSCs was relatively higher, but the interaction of *SP7* × tissue sources was still non-significant (*P* = 0.627). Correspondingly, compared with MSCs recovered from PR and IPFP, the expression of *osteocalcin* in OM and SC adipose-derived MSCs was higher, while the *osteocalcin* × tissue sources interaction remained non-significant (*P* = 0.951). Compared with all other tissue sources, the expression of *osteopontin* was higher in PR-derived MSCs as, but the *osteopontin* × tissue sources interaction remained non-significant (*P* = 0.995).

## Discussion

Various studies have reported isolation of canine MSCs from the adipose tissue, umbilical cord blood, Wharton’s jelly, and bone marrow [2, 3]. Adipose tissue is the most easily and readily available source for adult MSCs isolation. Most studies on the isolation of canine MSCs from adipose tissue have compared two or three different sources of adipose tissue [12, 13]. In our study, canine MSCs were isolated from four different adipose tissue sources (including SC, PR, OM, and IPFP) of the same animal. The cells from all four adipose sources fulfilled the criteria of MSCs [5]. The MSCs released after enzymatic digestion of adipose tissues from different origin exhibited typical fibroblast morphology, similar to those of AD-MSCs obtained from humans [14] and dogs [11]. IPFP-derived cells have a fibroblast-like morphology, but are relatively small, as previously reported by [4]. The proliferation potential among SC, PR, and OM-derived MSCs remained non-significant in our study and similar results have been reported in humans [14] and dogs [12]. However, the proliferation rate of IPFP-derived cells was significantly higher, which may be due to their smaller cell size [4, 13]. Vice versa, MTT assay demonstrated that the SC, PR, and OM-derived MSCs had higher cellular metabolism as compared to small-sized IPFP-MSCs. This response is directly proportional to the content of cell mitochondria and cell size [15]. On the 12th day of cellular propagation, the increase in IPFP-derived MSCs was associated with high mitochondrial biogenesis, which increased the number of cells [16]. All adipose-derived MSCs showed a positive expression of CD73, CD90, and CD105, which is in line with studies on MSCs isolated from canine [2, 4], bovine [17] and human adipose tissue [18]. In bovines, CD73 was more expressed in the Golgi apparatus and less on the cell membrane [17], but in our research, the expression of CD73 on cell membrane clearly indicates the difference in cell stage. CD73 acts as a signal and adhesion molecule to support cells anchoring in vivo and in vitro [19]. The expression of CD73 in AD-MSCs from all four tissue sources was further confirmed by flow cytometric and qPCR analysis, and our results are in line with previously reported studies in dogs [20]. Another study on canines reported reduced expression of CD73 [2] which is not in agreement with our study, maybe due to the sample collection method or different anatomical sites of tissue. CD90 is another important marker of MSCs, and plays a vital role in cell adhesion, migration, and cell-to-cell/−matrix interaction [21]. As previously reported in humans, CD90 is strongly expressed in all tissue-derived cells [22] at the same time, some studies in mice and dogs have reported abridged expression of CD90 [23, 24]. Therefore, the positive expression of CD90 on MSCs depends on the species, tissue source, passage number and culture conditions [4, 17, 23]. In this study, the expression of CD105 was relatively low compared with CD73 and CD90. While the expression of CD105 depends on the passage number and the site of tissue collection [20]. In murine, AD-MSCs with low CD105 expression may develop in the direction of adipocyte and osteocyte lineages [25]. CD45 is a cell surface tyrosine phosphatase that is present on hematopoietic origin cells containing the nucleus, including hematopoietic stem cells, but not on the non-hematopoietic origin cells. Therefore, CD45 is believed to be an elite marker of the hematopoietic lineage [2, 23]. Coherent with this fact, our study reported a very low expression of CD45 as compared to CD73, CD90, and CD105. In comparison to human MSCs, no standard criteria have been developed for the characterization of canine MSCs [26]. Such criteria are helpful because some contamination of culture with non-MSCs are inevitable during culture. Contamination with other cell types can be avoided or at least restricted by enhancing the culture density or repetitive media change and will decrease during passaging under optimized culture conditions [27]. It needs to be considered that the cells used in the present study represented a very early passage (P3). Nonetheless, these MSCs effectively differentiated into adipocytes with abundant cytoplasmic lipid droplets. Previous studies reported unsuccessful adipogenic differentiation of canine MSCs using human media [11], but our research method has efficiently differentiated canine MSCs into adipocytes. Compared with IPFP, the increase in ORO accumulation in OM-derived MSCs may be due to the increase in cell size. Apart from ORO concentration, the increased expression of *PPARγ, FABP4* and *FAS* genes also supports the MSCs differentiation into adipocytes.

The modified concentrations of dexamethasone, rosiglitazone, IBMX, and insulin worked very well in our experiments and have effectively differentiated MSCs into adipocytes within 7 days’ time. In contrast, [28] reported the loss of adipogenic differentiation due to the presence of IBMX in canine induction media. Our results are consistent with those of Ritter et al. [29] who pointed out that the adipogenesis of human-derived OM was higher than that of SC cells. Another study did not describe any difference in adipogenesis between OM and SC derived AD-MSCs [2], this response may be due to the use of low glucose medium in the current study. However, when we cultured MSCs in high glucose medium, our cells developed to the senescence stage (data not shown). Dexamethasone is used in both adipogenic and osteogenic differentiation of MSCs and in combination with IBMX, it can promote PPARγ-induced adipogenesis [30]. Rosiglitazone is also a PPARγ agonist, which accelerates the sensitivity of cells to insulin and promotes conversion into adipocytes. The adipogenic differentiation also requires insulin to initiate the transport of glucose, cholesterol, and fatty acids to the developing adipocytes. That’s why *PPARγ* is considered an early and *FABP4* as a late marker of adipogenesis based on *FABP4* involvement in the cellular transportation and metabolism of fatty acid [31]. An increased expression of *PPARγ* and *FABP4* mRNA was observed in OM and IPFP-originated cells, while the inconsistent qPCR results of SC and PR-derived MSCs may be due to the shortened time (7 days) provided in the differentiation medium. Even though the change remained non-significant, this difference may be due to the increased adipogenic potential of OM and IPFP-derived cells, or the co-incubation of cells with IBMX/rosiglitazone. The increase in *FAS* mRNA expression in differentiated cells from all sources indicates that the cells have a high demand for fatty acids and seems to be related to the potentiated action of insulin during adipocytes development [32].

Canine MSCs from all four sources were effectively differentiated into osteocytes and stained positively with ARS. During the process of osteogenesis, cells regularly detach from the surface and this phenomenon has already been reported by [4]. However, we did not observe any detachment in IPFP-derived cells during the osteogenesis process. Cells isolated from OM adipose tissue showed significantly higher ALP development, because differentiated cells express RUNT-related transcription factors that regulate osteogenic genes, including *ALP* and *osteocalcin* [33]. One of the early osteoblast differentiation factors is ALP [34]. Regardless of our tissue source, ALP was high in all differentiated cells. In our experiment, the gene expression of *osteopontin* (second early osteogenic marker) was non-significant to that of the control group. This response may be because we checked the expression 21 days after osteogenesis when gene expression of *osteopontin* may have been reduced [35]. Even though, the immunocytochemical positive expression of osteopontin indicates that the antigen exists outside the cell and plays an important role in the accumulation of hydroxyapatite minerals. *Osterix*/*SP7*, a zinc finger transcription factor, plays a key role in the differentiation of MSCs into osteoblasts [36]. Compared with control samples, osteogenic differentiated cells from all adipose tissue sources (SC, PR, OM, and IPFP) showed high levels of *SP7* and *osteocalcin*. The osteogenic differentiation induced by dexamethasone may increase the expression of *osteocalcin* through the formation of hydroxyapatite minerals in the presence of β-glycerophosphate [37]. In the process of mineralization, the importance of dexamethasone concentration for the gene activation of *osteocalcin* and osteogenic differentiation has also been reported [38]. Our findings indicated that, among all four sources, the OM and IPFP-derived MSCs have the better bi-lineage differentiation ability. On the other hand, IPFP derived MSCs have the highest proliferation rate among the studied sources of MSCs. Previous studies on canine reported the successful treatment of osteoarthritis using AD-MSCs and platelet-rich plasma (PRP) [39]. Similarly, the use of IPFP derived MSCs along with autologous PRP gels have shown promising results in the treatment of canine bone defects [40]. These studies provide solid evidence for the clinical application of AD-MSCs in canines. Many other factors including age, gender, cell isolation and differentiation protocols also affect the biological characteristics of stem cells [41]. A study on canine AD-MSCs reported an inverse correlation between cryopreservation and proliferation ability, but did not have a negative impact on the multilineage differentiation ability. In cell-based therapies, cell senescence is another important factor that MSCs show in higher passages. In higher passages, cells may become biologically exhausted, leading to poor proliferation and differentiation abilities [42]**.** Vice versa, another study conducted in our laboratory showed that freshly harvested autologous canine IPFP-MSCs showed significant proliferation rate and differentiation capacity, and they were successfully transplanted into critical sized bone defects [40, 43]. Overall, our present study provides a novel comparison for the isolation, characterization, and differentiation of canine AD-MSCs from different anatomical origins of the same animal.

### Conclusions

In conclusion, functional and molecular evidences suggest that canine OM adipose tissue is an excellent source for MSCs isolation and lineage differentiation. In addition, IPFP-based MSCs also have enhanced proliferation capacity and comparable adipogenic or osteogenic competencies. Our results suggest that, tissue source and proliferative potential should be considered an important aspect of stem cell therapy along with lineage differentiation abilities.

## Methods

### Tissue collection

Three severely injured dogs (6–10 months) were presented at the outdoor clinic of the Faculty of Veterinary and Animal Science, PMAS-Arid Agriculture University, Rawalpindi, Pakistan. After proper examination and on advice of the registered hospital veterinarian, the dogs were euthanized for reasons unrelated to this study. Euthanasia followed the American Veterinary Medical Association (AVMA) guidelines for euthanasia of animals [44]. Briefly, a deep stage of anesthesia was achieved with intramuscular injection of xylazine HCL (1 mg/kg) and ketamine (5 mg/kg), in which cardiac arrest was induced by an intravenous injection of supersaturated solution of MgSO_4_ (1 mL/kg). After euthanasia, adipose tissue (~ 3–5 g) from the inguinal subcutaneous (SC), omental (OM), and perirenal (PR) region and from the infrapatellar fat pad (IPFP) were isolated as presented in Fig. 1A-D, and kept in Dulbecco phosphate buffer saline (DPBS^−/−^, i.e. without Ca^++^ and Mg^++^; Sigma-Aldrich, USA) with 5% penicillin (100 U/mL), streptomycin (100 μg/mL) and amphotericin-B (250 μg/mL, Caisson, USA) solution and transported to Stem Cell Physiology and Cytogenetic Laboratory at Faculty of Veterinary and Animal Sciences, PMAS-Arid Agriculture University, Rawalpindi, Pakistan. All experimental procedures were performed in strict accordance with the guidelines of the Institutional Animal Ethics Committee.

### Cell isolation and culture

The adipose tissue sample was thoroughly washed with DPBS^−/−^ having 5% penicillin-streptomycin, amphotericin-B solution, and sliced to prepare a slurry. This suspension was digested with type I collagenase (0.1 mg/mL; Solarbio, China) in Dulbecco’s Modified Eagle’s Medium-Low Glucose (DMEM-LG, Bio West; France) at 37 °C for 135 min. The enzymatic activity of collagenase was stopped by adding an equal amount of LG-DMEM containing 10% fetal bovine serum (FBS; Bio West, France). The cells released during the digestion process were sieved through a 100 μm mesh (Corning, USA) and centrifuged at 548 *g* for 10 min at room temperature. The recovered cell pellet was resuspended in complete LG-DMEM (supplemented with 10% FBS, 1% penicillin-streptomycin and amphotericin-B) and seeded in T-25 tissue culture flasks. The cultured cells were incubated in a humidified environment at 37 °C and 5% CO_2_. The medium was changed every 48 h until the cells reach 80 to 90% confluence. The confluent cells were washed twice with DPBS^−/−^ and treated with a working solution of trypsin-EDTA (0.05% and 0.53 mM w/v, respectively; Caisson, USA) for 10 min in a humidified environment at 37 °C with 5% CO_2_, trypsin activity was halted with complete LG-DMEM. The recovered cells were centrifuged at 548 *g* for 5 min and sub-cultured till passage number two (P-2).

### Mesenchymal stem cells doubling time

The cell doubling times at P-3 was calculated on day 3, 6, 9, and 12. Briefly, 5000 cells/well were cultured in a 48-well cell culture plate. On each calculation day, the medium was removed, and the cells were washed twice with DPBS^−/−^. Subsequently, the cells were trypsinized as described in the previous subsection. Recovered cells were suspended in 1 ml of complete LG-DMEM and counted using a modified Neubauer chamber. Cell viability was assessed by the Trypan blue exclusion test (> 92%).

### Mesenchymal stem cells metabolic assay

The metabolic activity of MSCs was determined at 3, 6, 9, and 12 days of culture using the 3-(4, 5-dimethylthiazol-2-yl)-2, 5-diphenyltetrazolium bromide (MTT) dye. Briefly, 5000 cells/well were cultured in 48-well culture plates in complete LG-DMEM. The metabolic activity of the cells was evaluated by adding 0.25 μg/ml MTT dye, and incubated for 180 min at 37 °C in a humidified chamber containing 5% CO_2_. The assay measures the ability of cells to reduce MTT into blue formazan crystals with mitochondrial dehydrogenase enzyme. After removing the supernatant, the obtained formazan crystals were mixed into 100 μl of dimethyl sulfoxide (DMSO; Sigma-Aldrich, USA), and the absorbance was recorded at 630 nm using a microplate reader (BioTek 800TS, USA).

### Immunophenotyping of mesenchymal stem cells

Flow cytometric analysis was performed at P3 for quantitative expression of MSC antigens. Briefly, after trypsinization, 5 × 10^5^ cells were pelleted from the culture and resuspended in DPBS^−/−^. Polyclonal rabbit-raised primary antibodies against CD73 (E-AB-10944), CD90 (E-AB-16098), CD105 (E-AB-34276) (Elab Science, USA), diluted (1:100) in DPBS^−/−^ were added and incubated at 37 °C for 15 min. The cells were then washed with DPBS^−/−^ and centrifuged at 500 *g* for 5 min at room temperature to remove unconjugated antibodies. The secondary antibody (anti-IgG) conjugated with Alexa Flour-488 was diluted in DPBS^−/−^ (1:300) and added for incubation at 37 °C for 15 min (separate FACS tubes for each antigen). Afterwards, the cells were washed with DPBS^−/−^ (300 *g*) and processed for flow cytometry. For quantification of CD45 (ED7018, Exbio, Czech Republic), the mouse raised antibodies coupled with FITC (1:100) were used. The expression of cell surface markers was carried out by flow cytometry (FACScan, BD Biosciences, USA) using CELLQuest (BD Biosciences, USA) software.

In order to visually determine the antigens (CD73, CD90, CD105, FABP4 (E-AB-60028; Elab Science, USA) and osteopontin (ab63856, abcam, UK), the cells were grown on 6 mm round glass coverslips. After reaching 70% confluence, the cells were fixed with 4% buffered formalin for 20 min, washed with DPBS^−/−^, treated with 0.3% Triton X-100, and blocked with 10% goat serum. The cells were incubated with polyclonal rabbit-raised primary antibodies against CD73, CD90, CD105, FABP4, and osteopontin (diluted 1:50 in DPBS^−/−^) for 1 h at room temperature, and then overnight at 4 °C. Afterwards, the cells were washed with DPBS^−/−^ and incubated with Alexa Flour 594-conjugated secondary antibody (1:400) at room temperature in the dark for 45 min. Next, cells were washed twice with DPBS^−/−^ and incubated with 0.2 μg/mL of DAPI stain for 5 min in the dark. Cells were thoroughly washed with DPBS^−/−^, and mounted to the glass slides with an anti-fading mounting media (Vecta shield, UK). The fluorescence signal was evaluated on an epi-fluorescence microscope (Leica DMI 6000B, 63× objective).

### Bi-linage MSCs differentiation assay

At P3, in vitro differentiation of MSCs was performed into adipocytes and osteocytes. For the adipogenic differentiation assay, 2.5 × 10^4^ cells/well were seeded in 24-well plates for confluence. Cells were provided with an adipogenic induction medium that was LG-DMEM supplemented with 10% FBS, 0.1 mM IBMX (Sigma-Aldrich, USA), 10 μM rosiglitazone, 0.3 mM dexamethasone, 5 μg/mL insulin (Nova Nordisk, Denmark), and 1% penicillin-streptomycin, and amphotericin-B. After 48 h, the adipogenic induction medium was replaced with adipogenic maintenance medium, which composed of LG-DMEM supplemented with 1% Ex-cyte (Millipore, USA), 5 μg/mL insulin, 1% penicillin-streptomycin, and amphotericin-B for 7 days with medium change after every 48 h. Before staining with oil-red-O (ORO), the cells were washed with DPBS^−/−^ and fixed with 4% buffered formalin (Sigma-Aldrich, USA) at room temperature for 30 min and rinsed twice with DPBS^−/−^ to remove the traces of formalin. Subsequently, the working solution of ORO (6:4) was poured into all the wells and incubated in the dark at room temperature for 30 min. Cells were rinsed with DPBS^−/−^ and visualized under an inverted light microscope. The ORO stain was eluted from the cells using anhydrous isopropanol, the absorbance measured at 490 nm and normalized as follow:

$$\mathrm{ORO}\ \mathrm{Quantification}=\mathrm{ORO}\ \mathrm{Concentration}/\mathrm{Cell}\ \mathrm{Number}\times \kern0.37em 100$$

For osteogenic differentiation, 2.5 × 10^4^ cells/well were seeded in 24-well plates. After reaching confluence, the cells were provided with an osteogenic medium consisting of α-MEM supplemented with 10% FBS, 10 mM β-glycerophosphate (Sigma-Aldrich, USA), 50 μM ascorbate-2-phosphate, 100 nM dexamethasone, 0.75 nM vitamin-D_3_, 1% penicillin-streptomycin and amphotericin-B. After 21 days of incubation, the cells were stained with Alizarin Red Stain (ARS; Sigma-Aldrich, USA) to assess the degree of mineralization. Briefly, the medium was removed, and the wells were washed twice with DPBS^−/−^. The cells were fixed with 4% formalin for 30 min, and washed again with DPBS^−/−^. Then, ARS working solution (40 mM) was added and cells were incubated for 45 min in the dark. The dye was removed, cells were washed thrice with DPBS^−/−^ and observed under an inverted light microscope.

### Alkaline phosphatase activity (ALP)

After 21 days of osteogenesis, ALP activity was evaluated using a commercially available kit (ELITech Group, France) according to the manufacturer’s instructions. After cell lysis, p-nitrophenyl phosphate (p-NPP) was used as a substrate to detect ALP activity and normalized to the total protein present in the cell lysate detected using a commercially available protein assay kit (Bio-Rad, California, USA) following manufacturer instructions.

### Gene expression studies

Gene expression analysis was performed at the Institute of Veterinary Physiology, Freie Universität Berlin, Berlin, Germany. For this purpose, 1 × 10^5^ cells were seeded in a T-25 cell culture flask. After confluence, the cells were differentiated into the adipogenic or osteogenic lineages. For RNA isolation, the cells were scraped from the cell culture flasks, pelleted (548 *g*, for 5 min), transferred to RNA-later (Solarbio, China), and preserved at − 20 °C. The total RNA extraction was performed by using the NucleoSpin® RNA kit (Macherey-Nagel GmbH & Co. KG, Germany) as per manufacturer’s instructions and quantified by using a Nanophotometer (Implen®, Munich, Germany). Further, the RNA was reverse transcribed using the iScriptc DNA synthesis kit (Bio-Rad, Munich, Germany) according to the manufacturer’s instructions. The gene-specific primer sets for *CD73 (NT5E)*, *CD90 (THY1)*, *CD105 (ENDOGLIN)*, fatty acid synthase (*FAS)*, fatty acid binding protein 4 (*FABP4)*, peroxisome proliferator activated receptor gamma (*PPAR-γ)*, osteopontin *(SPP1)*, osteocalcin *(BGLAP)*, and osterix (*SP7*) were used in this study (Table.1). The qPCR reaction was performed in iCycler (Thermo Scientific, Massachusetts, USA) with SYBR green master mix (Bio-Rad, Munich, Germany) and the conditions were: initial denaturation at 94 °C for 3 min followed by 40 cycles of denaturation at 94 °C for 15 s, primer annealing at 58 °C for 25 s and primer extension at 72 °C for 1 min and with a final hold at 72 °C for 1 min. All reactions were performed in triplicate while keeping the *GAPDH* as a housekeeping gene. The 2^-ΔΔCT^ method was performed [45] to determine relative expression of target genes where a pool of undifferentiated cells from P3 were used as calibrator.

**Table 1:** The given primer sequences used to amplify specific genes of the canine mesenchymal stem cells and differentiated adipocytes/osteocytes

Gene	Sense 5′ - 3′	Anti-sense 3′ - 5′	Amplicon size (bp)
*CD 73*	TTTGGGGAAACCTTTGACC	AGAGGCTCGTAACTGGGTACTC	116
*CD 90*	CGGCTTCACCACCAAGGACG	TCTGGGCCAGCAGGCTTATG	140
*CD 105*	CCTCAGTGCAAAGAAGAAT	CTTGGAAGATCAGTTTGGGG	89
*FAS*	GGCTGGAGCCGGCTACTGCC	ATTCAGGATGGTAGCGTACA	94
*FABP4*	CACCATTAAATCAGAAAGCACC	CCAGGACACCTCCATCTAAG	128
*PPARγ*	TAAAGAGCCTGAGAAAGCC	GCTTCACATTCAGCAAACC	156
*Osterix*	TGCTTGAGGAGGAAGCTCAC	TTTGGGGGCTGAAAGGTCAC	161
*Osteocalcin*	TGCAACCTTCGTGTCCAAG	TGGAAGCCAATGTGGTCAG	171
*Osteopontin*	TGATTTTCCCACTGACATTCC	TCCATACTCGCACTTTTCAC	195
*GAPDH*	AAGAAGGTAGTGAAGCAGG	GCGTCGAAGGTGGAAGAGTGGG	212

## Statistical analysis

The data sets presented in this manuscript were statistically analyzed and graphs were plotted by using Sigma Plot 12.0 software (Systat Software Inc., San Jose, CA, USA). The experiment was performed in duplicate with three different animals, each of which served as an experimental unit. The observations of doubling time, ORO quantification, ALP activity, and flow cytometric analysis were arithmetically pooled from 2 wells of 6-well, 24-well, or 48-well cell culture plate where appropriate. The qPCR was conducted in triplicate. The gene expression of *CD73, CD90,* and *CD105* was analyzed by using one-way ANOVA. At the same time, all other data (doubling time/metabolic assay, ORO quantification, ALP activity, adipogenic, and osteogenic gene expression) were analyzed by two-way ANOVA, in which the interaction was maintained in the form of tissue × individual determinations. The significance among different groups was identified by Holm-Sidak post-hoc test. The data are expressed as mean ± standard error of the mean (SEM), where *P* < 0.05 is considered statistically significant.

## Data Availability

The datasets used and/or analyzed during the current study are available from the corresponding author on reasonable request.

## Abbreviations

MSCs: Mesenchymal stem cells
AD-MSCs: Adipose derived MSCs
BMSCs: Bone marrow derived MSC
SC: Inguinal subcutaneous
PR: Perirenal
OM: Omental
IPFP: Infrapatellar fat pad
DMEM-LG: Dulbecco’s Modified Eagle’s Medium-Low Glucose
Alpha-MEM: Minimum essential medium-alpha
FBS: Fetal bovine serum
DMSO: Dimethyl sulfoxide
IBMX: Isobutylmethylxanthine
DPBS: Dulbecco phosphate buffer saline
ORO: oil-red-O
ARS: Alizarin red stain
ALP: Alkaline Phosphates activity
FAS: Fatty acid synthase
FABP4: Fatty acid binding protein 4
PPAR-γ: Peroxisome proliferator activated receptor gamma
